# Sulfated Hydrogels in Intervertebral Disc and Cartilage Research

**DOI:** 10.3390/cells10123568

**Published:** 2021-12-17

**Authors:** Emily Lazarus, Paola Bermudez-Lekerika, Daniel Farchione, Taylor Schofield, Sloan Howard, Iskender Mambetkadyrov, Mikkael Lamoca, Iris V. Rivero, Benjamin Gantenbein, Christopher L. Lewis, Karin Wuertz-Kozak

**Affiliations:** 1 Department of Industrial and Systems Engineering, Rochester Institute of Technology, Rochester, NY 14632, USA; enl7795@rit.edu (E.L.); ivreie@rit.edu (I.V.R.); 2 Tissue Engineering for Orthopaedics and Mechanobiology, Bone & Joint Program, Department for BioMedical Research (DBMR), Medical Faculty, University of Bern, CH-3008 Bern, Switzerland; paola.bermudez@dbmr.unibe.ch (P.B.-L.); benjamin.gantenbein@dbmr.unibe.ch (B.G.); 3 Department of Orthopaedic Surgery and Traumatology, Inselspital, University of Bern, CH-3010 Bern, Switzerland; 4 Inamori School of Engineering, Alfred University, Alfred, NY 14802, USA; DAF10@alfred.edu; 5 Department of Biomedical Engineering, Rochester Institute of Technology, Rochester, NY 14632, USA; tls5331@g.rit.edu (T.S.); snh9508@g.rit.edu (S.H.); im6520@g.rit.edu (I.M.); mil9630@g.rit.edu (M.L.); 6 Department of Manufacturing and Mechanical Engineering Technology, Rochester Institute of Technology, Rochester, NY 14632, USA; cllmet@rit.edu; 7 Schoen Clinic Munich Harlaching, Spine Center, Academic Teaching Hospital and Spine Research Institute of the Paracelsus Medical University Salzburg (AU), 81547 Munich, Germany

**Keywords:** chondroitin sulfate, heparan sulfate, sulfated hyaluronan, sulfated alginate, sustained release of growth factors, chondrogenic phenotype

## Abstract

Hydrogels are commonly used for the 3D culture of musculoskeletal cells. Sulfated hydrogels, which have seen a growing interest over the past years, provide a microenvironment that help maintain the phenotype of chondrocytes and chondrocyte-like cells and can be used for sustained delivery of growth factors and other drugs. Sulfated hydrogels are hence valuable tools to improve cartilage and intervertebral disc tissue engineering. To further advance the utilization of these hydrogels, we identify and summarize the current knowledge about different sulfated hydrogels, highlight their beneficial effects in cartilage and disc research, and review the biofabrication processes most suitable to secure best quality assurance through deposition fidelity, repeatability, and attainment of biocompatible morphologies.

## 1. Introduction

Hydrogels are three-dimensional (3D), chemically or physically crosslinked polymer networks with hydrophilic groups that allow for high water-absorbing capacity. Their high water content results in physicochemical characteristics that are comparable to many tissues, including the intervertebral disc (IVD) and cartilage, thus constituting promising biomaterials for tissue repair and regeneration [1].

Furthermore, hydrogels are commonly used as drug delivery systems by providing spatial and temporal control over the release of therapeutics, predominantly via diffusion [2]; although, the diffusion rate depends on the selected polymer and can be further modified through, e.g., changes in water content, crosslinking density, porosity, and selection of a drug with a suitable diffusion coefficient, diffusion-dominated drug release is typically limited to hours or (at best) days.

Although synthetic and natural hydrogels are heavily researched, natural hydrogels such as hyaluronan, heparin, and alginate are commonly favored due to their biocompatibility, biodegradability, non-immunogenicity, and overall resemblance to the extracellular matrix (ECM) of connective tissues. However, despite their similarity to the IVD and cartilage, these hydrogels lack one crucial feature that originates from the high proteoglycan content of these tissues: the presence of carboxyl and sulfonic acid groups that create the IVD/cartilage-specific negatively charged ECM microenvironment [3]. Most recently, a study by Yang et al. demonstrated that the introduction of carboxyl groups into hydrogels and the resulting negative charges enhanced the chondrogenic differentiation of mesenchymal stem cells (MSCs) in vitro and in vivo [4]. However, as most published research thus far has used sulfonic acid groups to simulate a negatively charged microenvironment, this review paper will concentrate on sulfated hydrogels in disc and cartilage research and will focus on two beneficial effects arising from sulfation.

Firstly, the negative charges of sulfated hydrogels provide chemical cues to embedded cells that help induce and maintain a chondrogenic/disc-like phenotype and thus complement other signals arising from the simulated natural environment of biomimetic materials (e.g., mechanical cues). Details throughout this article demonstrate that 3D culture models based on sulfated hydrogels are superior to non-sulfated hydrogels and outperform classical 2D culture models. Phenotypic alterations of chondrocytes and IVD cells in vitro, a process also commonly termed dedifferentiation, involve increased expression of the fibroblast marker collagen type I and a concomitant reduction in the expression of collagen type II and proteoglycans [5].

Secondly, the negative charges of sulfated hydrogels furthermore offer the possibility to create a sustained release of positively charged therapeutic agents through electrostatic interaction [6,7] and have hence gained increasing interest in the drug delivery community over the past decade. So far, mainly growth factors have been incorporated into sulfated hydrogels. These diffusible signaling proteins stimulate cell growth, differentiation, and survival and modulate and control inflammation and tissue repair [8]. Although growth factors are potent modulators of cell behavior and thus have tremendous therapeutic potential, their clinical effectiveness is limited by their short half-life due to low stability, rapid internalization, and fast degradation [8]. Because of their positive charge, incorporating growth factors into sulfated hydrogels may protect them from degradation and inactivation and thus provide significant clinical benefits. Although less extensively investigated thus far, sulfated hydrogels can also be used to incorporate positively charged small molecules with, e.g., anti-inflammatory properties (for further information, see heparan sulfate).

**Figure 1:**
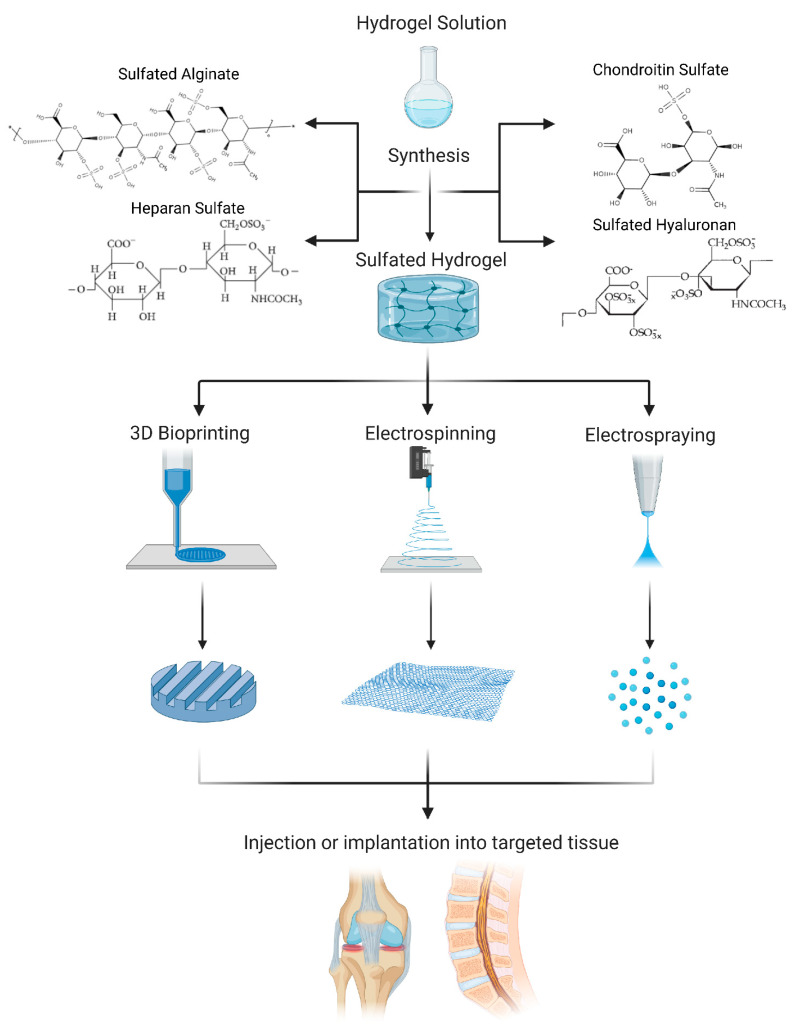
Schematic of the biofabrication process of sulfated hydrogels.

This review summarizes the state-of-the-art of sulfated hydrogels used in IVD and cartilage research. First, we focus on demonstrating their suitability as drug delivery systems (Table 1), with sustained release of growth factors and other positively charged drugs due to electrostatic interactions, hydrogen bonding, and other types of interactions [9]. Furthermore, we show their suitability to support a chondrogenic cell phenotype commonly lost during in vitro culture (Table 2), which is also relevant for research on the chondrocyte-like nucleus pulposus (NP) cells, i.e., the cells from the inner region of the IVD. Lastly, we highlight practical biofabrication techniques for sulfated hydrogels (Figure 1).

## 2. Sulfated Hydrogels Used in IVD and Cartilage Research: Overview

Over the past years, sulfated hydrogels have become increasingly popular in the cartilage and IVD research community due to their ability to support the chondrogenic phenotype and their potential for sustained drug delivery, as described in more detail below. However, their use either requires isolation from natural tissues or synthesis in the laboratory. While chondroitin sulfate (CS) and (to a lesser degree) heparan sulfate (HS) can be isolated from tissue, alginate, hyaluronan, and heparin are commonly used as base materials for the introduction of sulfate groups. Sulfated alginate is typically synthesized through the reaction of chlorosulfonic acid in formamide, whereby the degree of sulfation can be reproducibly tuned by altering the chlorosulfonic acid concentration [24]. Sulfation of hyaluronan is accomplished by reacting hyaluronic acid with an SO_3_/DMF complex.

Moreover, if sodium hyaluronate is first chemically modified to include methacrylate groups prior to sulfation, free-radical initiated polymerization of the resulting sulfated hyaluronan macromer forms a crosslinked hydrogel [7]. In a like manner, photo-crosslinkable hydrogels based on a methacrylated heparan sulfate macromers have also been reported. These are prepared by reacting heparan sodium salt with N-hydroxysuccinimide and 1-ethyl-3-(3-dimethylaminopropyl)-carbodiimide in a 2-(N-Morpholino)ethanesulfonic buffer for 5 min, followed by the addition of 2-aminoethyl methacrylate [15].

## 3. Chondroitin Sulfate (CS)

CS is an anionic linear polysaccharide composed of sulfated disaccharide repeating units with 1–3 D-glucuronic acid and N-acetylgalactosamine linkages. It is most commonly found in connective tissue throughout the body, including the IVD and cartilage [25]. It thus shows the potential for strategies targeting their repair and regeneration. One advantage of CS involves its mucoadhesive properties, which are due to the presence of hydroxyl groups. These functional groups can interact with and bind to the cell membrane through hydrogen bonding, thus creating adhesive binding sites between the hydrogel and cell membrane [17]. Other advantages of CS can be attributed to its high potency for cartilage deposition, which has also been shown to promote absorption of nutrients [15] and chondrocyte metabolism within the implanted hydrogel scaffold [14]. As such, biomimetic scaffolds based on CS have shown great potential in driving chondrogenic differentiation of MSCs by replicating the negatively charged ECM microenvironment of cartilage [14,15]. However, although negative charges are a crucial cue that impacts a biomaterial’s hydrophilicity, protein diffusion/binding, and cell adhesion/spreading [4], other aspects of the scaffold also determine its biomimetic capabilities, including biomechanical cues [26]. Wang et al. demonstrated optimal chondrogenesis of MSCs in CS methacrylate scaffolds with low mechanical stiffness around 7.5 kPa [15]. However, this mechanical instability can inhibit total integration with the local cartilage/IVD tissue, despite its mucoadhesive characteristics. Furthermore, as a glycosaminoglycan (GAG)-derived hydrogel, CS is characterized by fast degradation kinetics arising from hydrolytic and endogenous enzymatic mechanisms, thus increasing the risk for structural defects in the regenerating tissues. However, the enzymes responsible for CS degradation during cartilage defects are still unclear [14]. Extensive research is currently undertaken to circumvent these issues, e.g., by incorporating natural fibers and nanotubes to reinforce hydrogels, or adjusting the degradation time by creating photo-crosslinkable hybrid hydrogels [27].

Thus far, the use of CS for drug delivery has predominantly focused on nano-/microparticles (or composites thereof). Different approaches have been followed, including loading the drug into CS particles, coating particles of other origin with CS (with the drug being incorporated in the coating), and drug-loaded particles of other origin immersed in a CS hydrogel (Table 1). Following the latter approach, Fan et al. embedded chitosan-based microspheres (loaded with bovine serum albumin, as a model protein) into oxidized CS hydrogels supplemented with carboxymethyl chitosan. Compared to a hydrogel carrier without chitosan-based microspheres, the proliferation of chondrocytes was enhanced in the sulfated hydrogel carrier, probably due to enhanced chemical and physical similarity to the cartilage ECM. Furthermore, a more sustained BSA release was achieved using this composite (30% release after two weeks) compared to non-embedded microspheres (80%) or straight incorporation of serum albumin into the sulfated hydrogel without microspheres (51%) [13]. Due to the high biocompatibility, suitable biodegradability, non-immunogenicity, non-toxicity, and versatility, CS-based delivery systems will likely see a rising interest in cartilage and IVD research in the years to come, similar to the recent attention they have received for cancer therapy [28].

Recent studies have demonstrated the suitability of (functionalized) CS hydrogels to provide phenotypic support of chondrocytes and ultimately improve cartilaginous ECM accumulation. For instance, catechol chemistry inspired by marine mussels has been widely applied in tissue engineering due to its ability to form covalent crosslinks on the surface of any material, even wet tissue. Moreover, a catechol-functionalized CS-hydrogel was shown to create a native cartilage-like microenvironment and provide chondro-inductive signals, thus enhancing GAG deposition as well as increasing chondrogenic markers such as SOX9 and collagen type II in human adipose stem cells [14]. Similarly, soft methacrylate-CS hydrogels (~7.5 kPa) with encapsulated mesenchymal stem cells (MSCs) seemed to be optimal for the upregulation of aggrecan, collagen type I, collagen type II, and collagen type X and downregulation of MMP13 gene expression compared to the same hydrogels with higher matrix stiffness (~36 kPa). Overall, soft methacrylate-CS hydrogels seem to provide an anabolic (and less catabolic) environment, which is further evidenced by higher neocartilage ECM accumulation with 2.3 and 2.0 times higher deposition of sulfated GAG and collagen (compared with the hydrogel control) [15]. Aside from using pure CS-hydrogels, researchers also work on creating composites with other biomaterials. Initial studies on pullulan-based injectable hydrogels found that the CS-tyramine (CS-TA) content in pullulan-tyramine (CMP-TA) and CS-TA hydrogel systems directly affected gene and protein expression of chondrogenic markers including aggrecan, collagen type I and collagen type II, cell proliferation, and accumulation of total collagen in the ECM in primary chondrocytes. More specifically, a 3:1 ratio of CMP-TA/CS-TA was considered the most promising hydrogel system for mimicking the cartilage microenvironment and maintaining the chondrocytes phenotype, with significantly higher gene and protein expression of aggrecan, collagen type I, and collagen type II [16].

CS-based hydrogels have also been investigated as a potential 3D system for IVD regeneration. For example, in a recent study conducted by Borrelli et al. (2020), an injectable biomaterial comprising functionalized CS and decellularized ECM from bovine NP tissue was developed. Interestingly, the presence of CS in this biomaterial appears to be essential to promote higher amounts of sulfated GAG deposition [18]. In addition, a previous study on injectable PNIPAAm-g-CS copolymers for NP tissue engineering showed cytocompatibility of this biomaterial with human embryonic kidney 293 cells [17].

## 4. Heparan Sulfate (HS)

HS is a linear polysaccharide composed of repeating disaccharides of 1,4-linked uronic acid and glucosamine residues [25]. HS is found in cartilage and the IVD. More importantly, together with CS, it is one of the main contributors of negative charge and hydration to the ECM [22]. Although it can be isolated from tissues, it is most commonly sulfated synthetically from heparin due to its ability to tailor its mechanical properties and degradation time. Nonetheless, HS is characterized by fast degradation, limiting its usability (e.g., for tissue engineering) and requiring similar solutions to extend its stability in vivo, as discussed for CS [29].

Despite the fast degradation and the potential need for modifications, HS is highly sulfated and consequently has a strong binding affinity to growth factors from the FGF and TGF families (Table 1), thus, making this hydrogel an excellent growth factor reservoir [15,30]. Furthermore, HS hydrogels can also prevent growth factors from enzymatic degradation, thus potentiating their biological activity [31]. HS has also been used as a release system for the anti-inflammatory drug Crystal Violet due to its electrostatic interactions, which lead to a very constant rate of release with almost zero-order kinetics [30]. Although the use of HS and other sulfated hydrogels to deliver anti-inflammatory drugs is still evolving, their general characteristics are promising to further support this application in the future.

Due to their high degree of sulfation, heparin-based hydrogels have been widely studied in cartilage tissue regeneration [25]. These studies have demonstrated their ability to support the chondrogenic phenotype and promote the production of cartilage ECM deposition typically lost during in vitro cultures. For example, chondrogenic gene expression markers and homogeneous ECM deposition were upregulated in isolated bovine chondrocytes encapsulated in horseradish peroxidase crosslinked dextran–tyramine (Dex–TA)/heparin–tyramine (Hep-TA) composite hydrogels compared to Dex-TA hydrogels [19]. Moreover, Dex-TA hydrogel had an abundance in collagen, implying chondrogenesis promotion with the addition of Hep-TA. Wang et al. also reported an increase in collagen type II and aggrecan expression in HS-methacrylate hydrogels encapsulating human MSCs. Compared to the control polyethylene glycol (PEG) hydrogel, both soft (~7.5 kPa) and stiff (~36 kPa) HS-methacrylate hydrogels demonstrated an increase in collagen type II and aggrecan gene expression [15]. Lastly, HS has also been combined with scaffolds to reinforce cartilage regeneration. This was demonstrated by Kim et al. and their constructed gelatin incorporated poly(L-lactide-co-ε-caprolactone) (PLCL) scaffold with thiol derivative of heparin (Hep-SH) hydrogel for partial-thickness cartilage regeneration. In vitro, chondrocytes derived from rabbit articular cartilage encapsulated in the PLCL scaffold Hep-SH hydrogel system demonstrated enhanced expression of chondrogenic genes and the promotion of GAG deposition compared to the PLCL control scaffold. This was supported by chondrocyte differentiation demonstrated with an increase in collagen type II gene expression and decreasing collagen type I. Similarly, collagen type II, as well as aggrecan and GAG deposition, were more abundant, while collagen type I was scarce in PLCL scaffold/Hep-SH hydrogel implanted in vivo rabbits compared to control [20]. Overall, these results suggest an increase in chondrogenic phenotypic support with sulfation.

## 5. Sulfated Alginate (SA)

SA is created through modification of alginate, a natural polysaccharide extracted from algae that consists of linear chain structures of 1–4 linked mannuronic acid (M) and l-guluronic acid (G). Alginate has been widely used in biomedical research due to its high biocompatibility, low toxicity, low cost, ease of gelation, structural similarity to the ECM, and ability to modify and control drug release through alterations of the crosslinking process [32]. Alginate-based hydrogels have thus been contemplated as the golden standard for tissue engineering research and are one of the most commonly employed hydrogels for synthetic sulfation. However, previous pre-clinical and clinical studies have shown a risk for calcification of alginate, especially when implanted in vivo [33,34,35], which can negatively affect soft tissue regeneration, including cartilage and disc-like tissue. In contrast, recent research highlights that SA hydrogels may suppress hypertrophic calcification due to the repulsion between negative changed sulfate and phosphate groups [36]. In addition, SA mimics the proteoglycans found in native tissue, promotes chondrogenesis, enhances angiogenic activity, and allows encapsulation, retention, and sustained release of molecules, including growth factors [10,37]. Although SA shows excellent biological properties, the sulfation process negatively affects its physicomechanical properties, resulting in decreased stabilization and lowered mechanical stiffness (due to increased swelling rates). The sulfation process was shown to decrease the compression modulus from 44.4 ± 3.21 kPa to 2.4 ± 0.57 kPa for 2% alginate hydrogels [21].

These changes will inherently alter the mechanical cues arising from the SA scaffold, which in turn may affect MSC differentiation [38]. However, as existing data on optimal matrix elasticity/stiffness for chondrogenic differentiation or maintenance of a chondrogenic phenotype are highly conflicting [39], future research will be needed to investigate whether an increase in the mechanical strength of SA hydrogels (e.g., by using alginates with a low M/G ratio [40]) will better promote chondrogenic gene/protein expression. Nonetheless, it is clear that for load-bearing tissues such as cartilage and the IVD, these inferior mechanical properties may restrict the in vivo use of SA. To overcome this limitation, the use of composites is promising. Research has shown that the addition of electrospun mats composed of polycaprolactone acts as a reinforcement thus improving the mechanical properties of the SA [41]. Furthermore, supplementing alginate with hyaluronan was shown to result in stronger hydrogels while also improving cell–biomaterial interaction and thus driving chondrogenic differentiation of MSCs [42], and the same may be true for SA.

Despite these challenges, SA has been used widely for the culture of chondrocytes, with the overall goal to facilitate cartilage regeneration. Multiple papers have shown that SA generally increases cell proliferation, cell adhesion, and collagen type II expression, which creates a more hospitable physiological environment for chondrocytes. For example, bovine chondrocytes embedded in calcium-crosslinked SA strongly increased cell proliferation and RhoA activity when compared to non-modified alginate [21]. These findings also suggest that the RhoA GTPase pathway is essential in modulating chondrocyte proliferation through the upregulation of Cyclin D1. Aside from classical calcium-induced crosslinking, different alternative crosslinking approaches have been tested for SA. SA crosslinked with tyrosinase caused increased SOX9 expression and provided strong chondroprotective properties in the long term, with a 50-fold decrease in collagen type I expression after 21 days [22]. Barium-based crosslinking of SA resulted in decreased expression of Sef, a modulator of FGF signal transduction, which has been shown to regulate osteogenesis but has no known expression and regulating factors in chondrocytes [10]. These results indicate that the sulfation itself and the method of crosslinking can affect cell behavior. Crosslinking methods and their impact on phenotypic support should therefore be investigated in more detail and will have to be considered in future clinical applications.

SA has furthermore been extensively used for the delivery of fibroblast growth factor (FGF) but can also be applied to other drugs and growth factors. For example, chondrocytes were co-embedded with FGF in SA with different sulfation degrees (none, low, and high) to determine FGF signaling-mediated proliferation and ECM synthesis dependent on FGF release profiles. Highly sulfated alginate (high-SA) had higher initial FGF2 encapsulation (as demonstrated by significantly increased proliferation), as well as better FGF2 retention. Notably, after two weeks, high-SA still contained about 40% of the initially loaded FGF2, while only 10–20% were maintained in the control hydrogels, resulting in significantly more collagen type II and proteoglycan deposition with high-SA [10]. In support of this, Freeman et al. showed that SA could be successfully used to control the release of basic FGF (bFGF), whereby variations of the amount of sulfation and thus the amount of bound bFGF allow for release tunability [37].

## 6. Sulfated Hyaluronan (SH)

SH is based on hyaluronan, a high molecular weight GAG composed of a linear polysaccharide with disaccharide repeats of D-glucuronic acid and N-acetyl-D-glucosamine. It has been widely used for drug delivery, tissue engineering applications, and viscosupplementation due to its biocompatibility, non-immunogenic, and shock-absorption properties [43]. However, hyaluronan hydrogels experience fast degradation in vivo by enzymes such as hyaluronidase and do not allow for adequate cell adhesion [11]. The sulfation of hyaluronan has been investigated to overcome these limitations. Additionally, SH promotes chondrogenesis due to the increased binding affinity of growth factors, as described in more detail below. Although SH shows improved biological properties, a disadvantage of SH is the possibility of high anticoagulant activity if the degree of sulfation exceeds 2.5 [11].

Different types of methacrylated and, thus, photo-crosslinkable SH have been investigated for drug delivery. SH has previously been used to promote wound healing through the incorporation of Epidermal Growth Factor (EGF), whereby hyaluronan/collagen-based hydrogels supplemented with acrylated SH proved to have a prolonged EGF release and increased effectiveness on dermal fibroblasts when compared to hydrogels that did not contain hyaluronan [11]. Notably, the EGF receptor signaling pathway also plays an essential role in cartilage development and homeostasis. Thus, it may constitute an attractive candidate for future studies in the IVD and cartilage field [44]. Aside from EGF, prolonged release of stromal cell-derived factor-1α (SDF-1α) [7] as well as transforming growth factor-β1 (TGF-β1) [12] could be achieved in SH due to the electrostatic interactions between sulfates and amino acids. In the latter study, the effectiveness of different sulfation degrees was tested. Results demonstrate that after seven days, hyaluronan hydrogels with low sulfation released 40% less TGF-β1 than non-sulfated hydrogels and that the drug retention could be further enhanced to 50% through increased sulfation degrees. The improved growth factor retention in the sulfated hydrogels suppressed the hypertrophy and enhanced the chondrogenesis of the encapsulated human MSCs in vitro, and -when injected in vivo - resulted in quality neocartilage tissue free of hypertrophic calcification [12]. Notably, chondrogenic markers including aggrecan and collagen type II were significantly upregulated in low and high sulfation hyaluronan hydrogels, while a lower expression of MMP13 and collagen type X hypertrophic markers and more uniform GAG deposition was reported. Of note, the cell viability of the encapsulated MSCs was not affected by the sulfation of the hydrogel.

MSCs have also been extensively investigated for the treatment of IVD degeneration, and results by Peroglio et al. showed that their differentiation into a disc-like phenotype could be achieved in vitro and ex vivo when incorporated into a hyaluronan-based thermoreversible hydrogel (HA-pNIPAM). Notably, NP cell re-differentiation in this hydrogel was supported, as evidenced by upregulation of aggrecan and downregulation of collagen type I as well as ECM accumulation [23]. Thus, the use of sulfated HA-pNIPAM may further improve the outcome of IVD tissue engineering and repair strategies due to the cues provided by the sulfation groups, but also because of the possibility to incorporate growth factors as signaling molecules.

## 7. Biofabrication Techniques

Aside from different casting methods and bead formation, biofabrication techniques are increasingly used for sulfated hydrogels as the clinical application of implantable scaffolds requires precise sizes and shapes to match that of the defected area. 3D bioprinting, electrospinning, and electrospraying are standard biofabrication techniques (Figure 1) used to produce scaffolds that mimic structural features of native tissue.

3D bioprinting is a layer-by-layer material deposition technique that allows for control over scaffold pore size, geometry, and porosity. These structural characteristics are essential for promoting cell viability, proliferation, differentiation, and migration. However, bioinks play an integral role in the deposition quality that can be achieved with this technique. For this purpose, researchers are investigating CS and SA bioinks for the fabrication of 3D bioprinted cartilage scaffolds. Abbadessa et al. 3D-printed methacrylated CS scaffolds with suitable mechanical and biological properties and tunable porosity to achieve chondrogenesis [45]. Due to CS lacking essential mechanical properties needed for 3D printing applications, methacrylated CS was mixed with a synthetic thermo-sensitive polymer, partially methacrylated pHPMAlac-PEG triblock copolymer, to improve the rheological profile [45]. 3D bioprinting has also been implemented for the fabrication of SA scaffolds for cartilage tissue engineering applications. Muller et al. successfully fabricated a SA bioink that supports the chondrocyte phenotype. However, due to the low yield point of alginate sulfate, Muller et al. added nanocellulose to increase mechanical stability when printing. Due to this “trick” the rheological behavior of the bioink increased from 0.05 Pa*s to 10.6 Pa*s. It should be pointed out that challenges with maintaining cell viability, proliferation, and spreading were present in 3D-bioprinted scaffolds fabricated with small diameter nozzles and valves [46].

Electrospinning is an electrohydrodynamic biofabrication method capable of yielding polymer-based scaffolds with high surface area to volume ratios and high porosity. The high surface area to volume ratios enhance the efficiency and release of loaded drugs and increase the scaffolds’ mechanical properties [47]. Daemi et al. developed 50wt% electrospun mats of SA combined with polyvinyl alcohol (PVA) using single nozzle electrospinning at a flow rate of 5 mL h^−1^. As compared to pure alginate, SA combined with PVA demonstrated improved electrospinnability [48]. Delgado-Rangel et al. investigated mechanical and biological properties of electrospun mats composed of collagen, PVA, CS, and hyaluronan and found improved mechanical properties of the citric acid crosslinked electrospun mats as compared to uncrosslinked mats. Of note, their pH-sensitive swelling behavior allows for an ideal drug delivery system, and a favorable cellular environment. Increases in CS content led to the production of spherical micro- and nanoparticles turning the electrospinning process into electrospraying. Although increases in CS reduced nanofiber spinnability, the high surface-to-volume ratio present with the production of spherical micro- and nanoparticles could improve cellular adhesions [49].

Lastly, the electrospraying of sulfated hydrogels can be used to fabricate nano- and microparticles as drug delivery systems for positively charged therapeutics. Due to their high fluid retention and absorption capacity, hydrogels allow for a sustained and controlled release of drugs. Researchers have successfully investigated the sustained and controlled drug release of therapeutics from alginate and CS electrosprayed microbeads. Khanal et al. encapsulated magnesium sulfate in alginate through electrospraying for Mg^2+^ delivery [50]. In addition to alginate, CS and PEG microparticles loaded with moxifloxacin have been successfully fabricated through electrospraying techniques. The encapsulation in CS and PEG creates a bioadhesive to allow for the localization of microparticle distribution through in situ gelling [51].

## 8. Conclusions and Outlook

Although sulfated hydrogels hold great promise in cartilage and IVD tissue engineering research concerning sustained drug release and phenotypic support, high costs and batch-to-batch variability associated with isolation from tissues are major limitations. Hence, extensive efforts have been made to create sulfated hydrogels in the laboratory, allowing for better control over sulfation degrees and tunability of their properties. Common limitations of sulfated hydrogels are inferior mechanical properties and fast degradation. With the ultimate goal of sulfated hydrogel research being clinical translation, future research will have to focus on circumventing these challenges. However, promising first approaches have already been developed, including the use of co-polymers or alternative crosslinking methods (including photo-crosslinking), as well as fiber- or nanotube-based reinforcement of sulfated hydrogels.

Furthermore, future activities will likely target the incorporation of positively charged therapeutics other than growth factors. Slow-release systems for Etanercept, a positively charged drug used to treat rheumatoid arthritis and other inflammatory diseases, could be investigated in the future. Other positively charged agents including Hydrocortisone (anti-inflammatory drug) and Lidocaine (anesthetic) have already been tested using different types of hydrogels and could next be embedded in sulfated hydrogels. Lidocaine, for example, is occasionally used for intra-articular injections but holds a high risk of toxicity that could be limited through controlled release by using sulfated hydrogels as a carrier material.

Lastly, upon development of improved sulfated hydrogels, their use as bioinks will require the identification of 3D bioprinting process parameters that help replicate the morphology of the envisioned tissues. As detailed before, electrospraying and electrospinning can be used to generate stand-alone structures. However, additional consideration should be made to incorporating electrospinning/-spraying in the process of 3D bioprinting to ultimately fabricate hybrid tissue engineering constructs for cartilage and IVD regeneration. Such constructs may hold promise to better address the mechanical and morphological characteristics of native cartilage and the IVD.

In summary, sulfated hydrogels are promising biomaterials that may help overcome some of the current limitations in cartilage and IVD tissue engineering, repair, and regeneration, especially if sophisticated (e.g., hybrid) biofabrication methods are employed.

## Figures and Tables

**Table 1:** Sulfated hydrogels for sustained release of growth factors.

Biomaterial	Cell Type	Growth Factor	Release	Ref
Sulfated Alginate Hydrogels				
Alginate sulfate hydrogels with high chlorosulfonic acid (ClSO_3_H) concentrations	Chondrocytes	FGF2	• Almost 40% of FGF2 retained after two weeks compared to the almost 20% retained by the controls	[10]
Sulfated Hyaluronan Hydrogels				
Sulfated hyaluronan (sHA)	Keratinocytes and dermal fibroblasts	EGF	• Prolonged release of EGF when studied over three days • Non sulfated hydrogels released a ten fold greater amount of EGF on day one when compared to sulfated hydrogel •Non sulfated hydrogel released a two fold greater amount on day three when compared to the sulfated hydrogel • Sulfated hydrogel had a fairly constant release rate	[11]
Sulfated HEMA-HA (HEMA-SHA)	N/A	SDF-1α	• Prolonged release when studied over twelve days • Sulfated hydrogel released the growth factor at ⅓ the rate compared to the non sulfated hydrogel control	[7]
LS-MeHA and HS-MeHA hydrogels	Human MSCs	TGF-β1	• Has extended release of TGF-β1 • 40–50% lower release amount when compared to non-sulfated hydrogel • Studied for days	[12]
Chondroitin Sulfate Hydrogels				
Chitosan-based microspheres (CMs) into CMC-OCS hydrogels	Chondrocytes	BSA	• Lowest release rate of BSA over two weeks • 30% of the sulfated hydrogel had released compared to 80% and 51% for the controls	[13]

Abbreviations: BSA, Bovine Serum Albumin; Human MSCs, Human Mesenchymal Stem Cells; TGF-β1, Transforming Growth Factor-β1; SDF-1α, Stromal Cell-Derived Factor-1α; EGF, Epidermal Growth Factor; FGF2, Fibroblast Growth Factor; HEMA-HA, Hydroxyethyl Methacrylate Hyaluronic Acid.

**Table 2:** Sulfated hydrogels for cartilage and IVD supporting phenotype with relevant gene expressions and cell responses.

Biomaterial	Cell type	Gene Expression(s)	Cell Response(s)	Ref
Chondroitin Sulfate Hydrogels				
Catechol-functionalized chondroitin sulfate	Human ADSCs	• Collagen type II and SOX9 ⬆ (compared to pellet culture)	• Good cell viability • Chondrogenesis ⬆ (compared to a pellet culture) • Significant GAG deposition • Good adhesion to cartilage tissue in vivo rabbit • Minimal loss of tissue in vivo rabbit • No significant pro-inflammatory cytokine secretion	[14]
Chondroitin sulfate methacrylate	Human MSCs	• Collagen type II and aggrecan ⬆ (in softer hydrogels) • Collagen type X ⬇ (in softer hydrogels) ^†^ • MMP13 ⬆ (in softer hydrogels) ^†^ • Collagen type I and MMP13 ⬆ (in stiffer hydrogels)	• GAG and collagen deposition ⬆ • Neocartilage deposition ⬆ (but decreases as stiffness increases) ^†^ • Homogeneous distribution of collagen type I & II with minimal collagen type X • Cellular remodeling observed	[15]
CMP-TA/CS-TA	Porcine auricular chondrocytes	• Collagen type I ⬇ • Collagen type II ⬆ • Aggrecan ⬆	• Cell viability and proliferation ⬆ • Highest collagen type II and aggrecan deposition with a 3/1 ratio • Fibrous tissue develop and no macroscopic sign of inflammation of toxicity in a rat model	[16]
PNIPAAm-g-CS	Human embryonic kidney 293 cells		• Low cytotoxicity • Good adhesive interphase with surrounding tissue	[17]
Bovine NP disc-derived self-assembled ECM functionalized with chondroitin sulfate	Porcine nasal tissue		• GAG/collagen ratio synthesis ⬆^‡^ • GAG deposition ⬆ • Collagen deposition ⬆ (predominantly collagen type II) • Rounded cell morphology	[18]
Heparin-based Hydrogels				
Heparan sulfate- methacrylate	Human MSCs	• Collagen type II and aggrecan ⬆ (in softer hydrogels) • High MMP13 expression (but decrease with increasing heparin sulfate concentration)	• Homogeneous distribution of collagen type I & II deposition • Collagen type X deposition ⬇ • Neocartilage deposition ⬆	[15]
HRP-crosslinked Hep-TA/Dex-TA	Bovine chondrocytes	• Collagen type II ⬆	• Cell viability ⬆ • Homogeneous distribution of collagen type II and CS deposition ⬆	[19]
Gelatin incorporated PLCL scaffold with Hep-SH	Rabbit articular cartilage chondrocytes	• Collagen type II ⬆ in vitro and in vivo rabbit model • Collagen type I ⬇ in vitro and in vivo rabbit model	• GAG deposition ⬆ • Collagen type II and aggrecan deposition in the scaffold ⬆	[20]
Sulfated Alginate Hydrogels				
Calcium-crosslinked sulfated alginate	Calf cartilage chondrocytes	• COL1A2/COL2A1 ratio ⬆^‡^ • SOX9/RUNX2 ratio ⬆^‡^	• Cell proliferation ⬆^†^ • RhoA activity ⬆^†^	[21]
Barium-crosslinked sulfated alginate	Bovine articular cartilage chondrocytes	• FGFR2 ⬇^‡^ • Sef ⬇^†^ • Collagen type II ⬆^‡^ • Aggrecan ⬇ • Collagen type I ⬇	• Cellular recognition/adhesion ⬆^†^ • Cell proliferation ⬆^†^ • FGF retention ⬆^†^ • Collagen type II deposition ⬆ • PG deposition ⬆ • Collagen type II ⬆ (in lower sulfation level) • PG deposition ⬆ (in higher sulfation level)	[10]
Tyrosinase-crosslinked alginate sulfate tyramine	Human and bovine articular cartilage chondrocytes	• Collagen type II ⬆^‡^ • Aggrecan ⬆^‡^ • Sox9 ⬇^‡^ • Collagen type I ⬇ • ADAMTS5 ⬇ • MMP13 ⬇	• Good cell viability • Demonstrates chondroprotective effects with FGF signalling • Collagen type I deposition ⬇ • Aggrecan deposition ⬆ • Fibrous capsule formation with cartilage specific matrix after 4 weeks in vivo mice	[22]
Sulfated Hyaluronan Hydrogels				
HA-pNIPAM	Bovine NP	• Collagen type I ⬇ • Aggrecan ⬆ • MMP13 and Has2 ⬆	• NP phenotype ⬆ • Normal cytocompatibility/viability • Generation of a NP cavity	[23]
Sulfated HA	hMSCs	• Collagen type II ⬆ • Aggrecan ⬆ • MMP13 ⬇ • Collagen type X ⬇	• Sulfation has no effect on cell viability • Uniform GAG deposition	[12]

Abbreviations: ADSCs, adipose-derived mesenchymal stem cells; CMP-TA, carboxymethyl pullulan tyramine; CS-TA, chondroitin sulfate-tyramine; Dex-TA, dextran–tyramine; ECM, extracellular membrane; GAG, Glycosaminoglycan; HA, Hyaluronanic acid; Has2, hyaluronan synthase 2; Hep-SH, Thiol derivative of heparin; Hep-TA, heparin–tyramine; HRP, horseradish peroxidase; MSCs, mesenchymal stem cells; NP, nucleus pulposus; Sef, similar expression to FGF genes; PG, proteoglycan; PLCL, poly(L-lactide-co-ε-caprolactone); PNIPAAm-g-CS, poly(N-isopropylacrylamide)-graft-chondroitin sulfate; pNIPAM, poly(N-isopropyl acrylamide); SOX9, SRY-Box transcription factor 9. ^†^ Compared with lower sulfation ^‡^ Compared to cells grown in a monolayer. “↑” means upregulation and ”↓” means down-regulation.

## Data Availability

Not applicable.
